# Biosurfactants in Food: Advances, Innovative Applications and Functional Perspectives

**DOI:** 10.3390/foods15030508

**Published:** 2026-02-01

**Authors:** Renata R. da Silva, Peterson F. F. da Silva, Carlos V. A. de Lima, Hozana de S. Ferreira, Jenyffer M. C. Guerra, Leonie A. Sarubbo, Juliana Moura de Luna

**Affiliations:** 1 Northeast Biotechnology Network (RENORBIO), Federal Rural University of Pernambuco, Dom Manuel de Medeiros Street, Dois Irmãos, Recife 52171-900, Brazil; renatabiology2015@gmail.com (R.R.d.S.); ferreira.hozanasouza@gmail.com (H.d.S.F.); 2 Department of Food Engineering, School of Food Engineering, University of Campinas, UNICAMP, Rua Monteiro Lobato, 80, Campinas 13083-862, Brazil; p254439@dac.unicamp.br; 3 Graduate Program in Fungal Biology, Department of Mycology, Federal University of Pernambuco, Professor Moraes Rego Avenue, Recife 50760-420, Brazil; carlosvinicius_adl@outlook.com; 4 Center for Technology and Geosciences, Post Graduate Program in Chemical Engineering, Department of Chemical Engineering, Federal University of Pernambuco (UFPE), Professor Moraes Rego Avenue, n. 1235, Cidade Universitária, Recife 50670-901, Brazil; jenyffer.campos@ufpe.br; 5 School of Technology and Communication, Catholic University of Pernambuco (UNICAP), Príncipe Street, n. 526, Boa Vista, Recife 50050-900, Brazil; leonie.sarubbo@unicap.br; 6 Advanced Institute of Technology and Innovation (IATI), Potira Street, n. 31-Prado, Recife 50070-280, Brazil; 7 School of Health and Life Sciences, Catholic University of Pernambuco (UNICAP), Príncipe Street, n. 526-Boa Vista, Recife 50050-900, Brazil

**Keywords:** microbial surfactants, food emulsifiers, clean-label, circular economy

## Abstract

Microbial biosurfactants have emerged as natural and sustainable alternatives to synthetic surfactants used in the food industry, due to the growing demand for biodegradable and safe ingredients. Produced by bacteria, fungi, and yeasts, these compounds exhibit important physicochemical properties, such as emulsifying capacity, surface tension reduction, foam stabilization, and favorable interaction with different food matrices. In addition to their technological function, they exhibit relevant biological activities, including antioxidant and antimicrobial action, which contribute to the control of lipid oxidation and microbiological deterioration. These characteristics make biosurfactants attractive for applications in emulsions, fermented beverages, aerated products, probiotic systems, and bioactive packaging. The objective of this work is to provide a narrative literature review that integrates recent advances in the production, functionality, safety, sustainability, and application perspectives of biosurfactants in the food sector. In the field of production, biotechnological advances have made it possible to overcome historical limitations such as high cost and low yield. Strategies such as the use of agro-industrial waste, metabolic engineering, microbial co-cultures, continuous fermentations, and in situ removal techniques have increased efficiency and reduced environmental impacts. Despite the advances, significant challenges remain. Future prospects and advances tend to facilitate industrial adoption and consolidate biosurfactants as strategic ingredients for the development of more sustainable, functional, and technologically advanced foods.

## 1. Introduction

The growing interest in surfactants has driven the search for molecules with lower toxicity and effective surface performance, attracting significant attention from both the scientific and industrial communities. Among the natural surfactants currently employed, egg yolk lecithin and milk proteins stand out, particularly in salad dressings and desserts, where they contribute to flavor, texture, appearance, and product stability. So-called “food additives” are substances added to foods with the purpose of preserving or improving characteristics such as flavor, texture, freshness, and appearance, in addition to ensuring food safety. However, it is essential that these additives be evaluated for their potential adverse effects on human health prior to application. Among the available options, biosurfactants emerge as highly versatile agents with potential for a wide range of applications [1].

The food industry has developed a wide variety of products, such as mayonnaise, salad creams, sauces, and desserts, using emulsifying compounds such as sorbitol esters, their ethoxylates, and sucrose esters. In addition, sulfonyl-based surfactants have been widely used in coatings, paints, and adhesives applied to different types of food packaging. Biosurfactants present significant advantages over synthetic surfactants, notably their biodegradability, low toxicity, potential for sustainable production, and broad functional versatility. These characteristics make them an environmentally conscious and suitable alternative for diverse industrial applications [2,3].

Based on their functional properties, the World Health Organization (WHO) and the Food and Agriculture Organization of the United Nations (FAO) have classified food additives into three main categories: (1) flavoring agents, (2) enzyme preparations, and (3) other additives. Within this context, microbially derived surfactants have gained prominence as versatile food additives capable of acting as emulsifiers, foaming agents, thickeners, texture enhancers, and preservatives. Furthermore, these molecules can be used for the encapsulation of lipophilic compounds, such as vitamins, contributing to their stability and bioavailability in foods. Beyond sustainability, the food industry can benefit substantially from encapsulation processes to protect active compounds, such as nutrients, enzymes, colorants, and probiotics, enhancing their stability and preserving their viability. This process helps prevent undesirable chemical reactions during storage and minimizes the risk of harmful substance formation [1,4,5].

In the food industry, biosurfactants stand out for their ability to form stable emulsions, making them useful as wetting agents, additives, and preservatives. Their potential application in this sector is associated with their effectiveness as emulsifiers, contributing to improved rheology, water retention, ingredient homogenization, and processing efficiency [6]. The production of emulsifiers through microbial cultures represents a promising alternative to conventional additives, enabling the development of more stable products aligned with modern food processing technologies. Studies report the use of biosurfactants as emulsifying agents in the processing of various raw materials, including meat products, where they assist in fat emulsification [4].

Biosurfactants such as rhamnolipids and sophorolipids have been incorporated into a wide variety of processed foods, including bread, hamburgers, baguettes, pizzas, croissants, salad dressings, cakes, cookies, and ice cream, with the aim of improving physicochemical stability as well as shape, structure, and texture properties [7].

Nevertheless, despite their application potential, biosurfactants are not yet used on a large scale as additives in the food industry. Moreover, their incorporation as new food ingredients depends on approval by competent regulatory agencies. In this context, the objective of this study is to analyze the potential of microbial biosurfactants as sustainable alternatives to synthetic surfactants used in the food industry, highlighting their functional properties, technological applications, advantages in terms of biodegradability and low toxicity, and their contributions to food product stability, quality, and safety. Additionally, this work distinguishes itself from previous publications by adopting a hybrid approach, combining bibliometric analysis with a critical narrative review, to demonstrate the technological maturity of the field. Uniquely, it provides an in-depth discussion on the challenges that still limit biosurfactants large-scale adoption, including regulatory, production, and incorporation aspects as novel food ingredients, identifying the specific gaps between GRAS status of microorganisms and the approval of purified molecules, a critical perspective often missing in general application reviews.

## 2. Materials and Methods

This study was conducted using a hybrid approach, combining quantitative bibliometric analysis with a narrative literature review, based on a comprehensive bibliographic survey of microbial biosurfactants with potential applications in the food sector. The narrative review was adopted because, according to Vosgerau and Romanowski (2014) [8], this type of review enables a broad analysis of the state of the art, encompassing scientific advances, technological limitations, and emerging perspectives.

Bibliographic prospecting was performed between October and December 2025, using the Scopus and Web of Science databases, considered primary sources of peer-reviewed articles. Searches were conducted using Boolean operators to combine descriptors in the title, abstract, and keyword fields. The main search query was structured as follows: ((“microbial surfactant” OR “biosurfactant”) AND (“food”) AND (“additive” OR “production” OR “antioxidant activity” OR “antimicrobial activity” OR “toxicology” OR “food safety” OR “regulation” OR “agro-industrial waste” OR “by-products”)). For the bibliometric mapping stage, a document type filter was applied, selecting original experimental articles and literature reviews, resulting in 566 documents.

These data were processed using R software (v. 4.3.1) with the Bibliometrix package (Biblioshiny) [9]. Prior to the bibliometric analysis, the datasets from Scopus and Web of Science were merged, and duplicates were automatically removed using the internal function of the Biblioshiny interface. Performance indicators and scientific mapping techniques were applied, including: (a) temporal evolution and annual growth rate; (b) technological life cycle; and (c) keyword co-occurrence networks.

Based on the bibliometric mapping, a qualitative screening was conducted to select publications for inclusion in the review. Studies published between 2017 and 2025 addressing functional properties in foods, biotechnological production and purification strategies, and safety and regulatory assessments were included. Studies focusing exclusively on bioremediation, petrochemicals, or lacking methodological clarity were excluded. It is worth noting that this review prioritized publications from 2017 to 2025, a period during which the most significant advances in the field were observed; however, earlier studies were included only when essential for contextualizing classical concepts.

At the end of the process, 122 scientific articles were selected for reading and critical analysis, supporting the discussion on functionality, safety, and innovative perspectives presented in this review.

## 3. Scientific Mapping and Field Evolution

**Figure 1:**
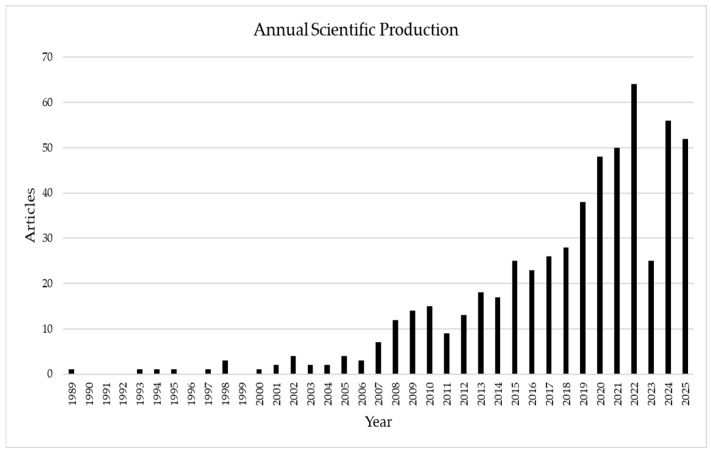
Temporal evolution of annual scientific production (1989–2025) referring to articles on microbial biosurfactants with potential application in the food industry. Data were obtained from systematic prospecting in the Scopus and Web of Science databases and processed using the Bibliometrix package (R). Source: Authors (2025).

Before delving into the technical analysis of biosurfactant applications, it is essential to understand the growth dynamics and maturity of this research field. Bibliometric analysis of the 566 retrieved articles reveals an accelerated expansion trajectory, particularly from 2018 onward (Figure 1). This phenomenon reflects not only biotechnological advances but also a scientific response to global demand for sustainable products.

The temporal evolution of experimental scientific production reveals three distinct phases of academic interest in biosurfactants within the food industry. Following a long latency period (1989–2006), sustained growth was observed from 2007 onward, culminating in a phase of accelerated expansion after 2018. The publication peak recorded in 2022 reflects the maturation of biotechnological routes and global pressure for clean-label and sustainable ingredients [10]. Despite a slight fluctuation in 2023, the last two years indicate renewed interest in the topic.

While annual production provides a quantitative overview of growth, understanding the technological maturity of this field requires an analysis of its evolutionary dynamics. To this end, the Scientific Production Life Cycle model was applied, using a logistic growth function to analyze the temporal trajectory of research and identify the current development stage of the area [11].

**Figure 2:**
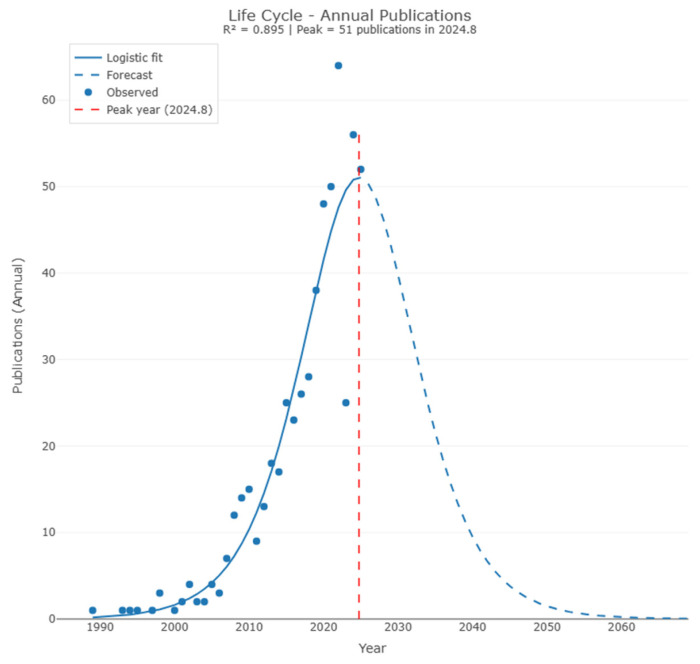
Logistic growth model and life cycle of scientific production on biosurfactants in foods. The curve (blue line) represents the mathematical fit, indicating the peak of technological maturity and saturation of scientific novelty in 2024. The projection (dashed line) estimates field stabilization as a consolidated technology.

The life cycle analysis reveals that experimental research on biosurfactants for food applications has reached technological maturity, with a robust logistic fit (R^2^ = 0.895), as shown in Figure 2.

The model identifies 2024 as the peak production year, suggesting that the field has transitioned from an exploratory discovery phase to a consolidation phase of innovative applications. This scenario justifies the relevance of the present narrative review, as the body of evidence accumulated up to 2025 provides a statistically robust basis for analyzing trends and sector limitations.

**Figure 3:**
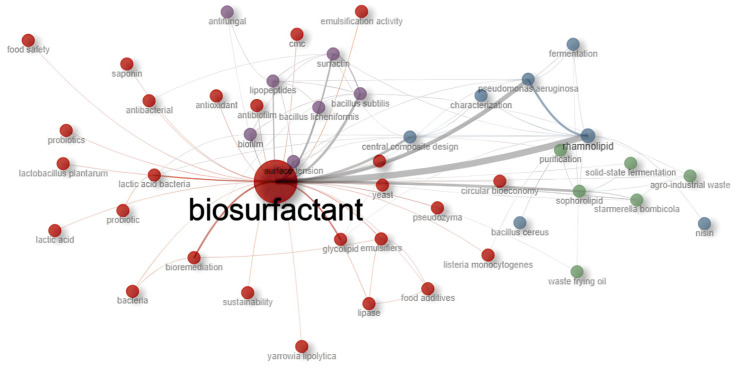
Keyword co-occurrence network based on 566 selected articles. Node size reflects term frequency, while colors indicate thematic clusters. Source: Authors (2025).

When crossing temporal maturity data with the keyword co-occurrence network (Figure 3), it is observed that the productive peak identified in 2024 is supported by significant thematic diversity. The network indicates that the field is no longer focused solely on biosurfactant production, but rather on their functionality within complex systems.

The grouping analysis identifies the drivers of this maturity as:

(1) Biological functionality and food safety (red group);
(2) Sustainability and circular economy (green group);
(3) Optimization and characterization (blue group);
(4) Biofilm control (purple group).

## 4. Recent Advances in Biosurfactant Production

Biosurfactants exhibit specific properties that are advantageous for food applications; however, their production cost remains relatively high, mainly due to raw material costs, fermentation efficiency (yield), and downstream purification processes. To overcome these challenges, recent research has focused on: (a) the use of agro-industrial residues as low-cost substrates; (b) genetic engineering to maximize yields or modify biological production systems; (c) co-culture strategies that promote metabolic synergy or induce biosurfactant synthesis; and (d) continuous or semi-continuous bioprocesses combined with In Situ Product Removal (ISPR), particularly via foam [12,13,14,15].

### 4.1. Agro-Industrial Residues as Substrates

The valorization of agro-industrial residues as substrates for biosurfactant production has emerged as a key strategy to reduce environmental liabilities and production costs of high value-added biomolecules [16]. Residues such as corn steep liquor, molasses, waste frying oil, peels and bagasse (rice, banana, sugarcane), and lignocellulosic materials have been extensively investigated as sources of carbon, nitrogen, and micronutrients for biosurfactant-producing microorganisms (Table 1). Recent reviews and experimental studies indicate that these substrates can partially or totally replace commercial media while maintaining productivity and functional performance of the final product [17].

The most commonly used substrates include sugarcane molasses, waste frying oil, corn steep liquor, and residues from fruit and grain processing [15,26]. These materials have been successfully employed by biosurfactant-producing microorganisms such as *Bacillus* spp. (lipopeptides), *Pseudomonas* spp. (rhamnolipids), and yeasts of the genera *Candida*, *Starmerella* (sophorolipids), and *Yarrowia*. Microbial adaptation to the residue and cultivation conditions are critical determinants of biosurfactant yield and composition [12,27].

The use of residues reduces operational costs and contributes to circular economy principles and environmental impact mitigation. When properly selected and processed, these substrates can achieve yields comparable to those obtained with synthetic media, especially when optimization strategies such as experimental design, mineral supplementation, and simple pre-treatments are employed [16,28].

However, compositional variability, the need for pre-treatment, the presence of inhibitory compounds, and difficulties in product recovery and purification still limit large-scale application, requiring the development of more efficient and economically viable extraction routes [29,30,31].

### 4.2. Genetic Engineering in Biosurfactant Production

Genetic engineering applied to strain improvement for biosurfactant production has advanced significantly, integrating transcriptional modulation, precision genome editing, and metabolic pathway optimization to increase yield, specificity, and cellular tolerance. Recent reviews emphasize the need to integrate metabolic engineering with process design to enable industrial-scale production [32,33,34,35].

In Bacillus subtilis, control of the srfA gene, combined with modifications in fatty acid biosynthesis pathways and fermentation optimization, has enhanced Surfactin production [36]. For Rhamnolipids, heterologous expression of the rhlAB genes in *Pseudomonas putida* has emerged as a promising alternative [37]. In addition, techniques such as MAGE (Multiplex Automated Genome Engineering) and recombineering enable simultaneous multiple genetic modifications, favoring biosurfactant production optimization [38].

High-precision genome editing tools, such as base editors and prime editors, allow targeted modifications in key regulatory elements and enzymes, reducing off-target mutations and increasing yield and genomic stability [32]. In oleaginous yeasts such as *Yarrowia lipolytica*, engineering of lipid metabolism and secretion systems has increased biosurfactant synthesis and excretion, particularly sophorolipids [39]. In parallel, genome reduction strategies and “lean chassis” (genetically simplified microorganism) approaches enhance metabolic predictability, requiring a balance between robustness and efficiency [40].

The integration of genetic editing with DBTL (Design-Build-Test-Learn) platforms, omics analyses, and genome-scale metabolic modeling has accelerated the prioritization of high impact genetic modifications, reducing experimental cycles and facilitating the transition from laboratory to pilot scale bioreactors [32,41,42].

### 4.3. Fermentation Processes for Biosurfactant Production

One of the main challenges for the economic viability of industrial scale biosurfactant production is fermentation process optimization. In this context, continuous and semi-continuous processes, as well as in situ product removal (ISPR) strategies, have gained prominence by increasing productivity, reducing purification costs, and mitigating inhibitory effects caused by product accumulation [14].

In continuous processes, constant substrate feeding and simultaneous broth withdrawal maintain cultures in a quasi-steady state, significantly increasing volumetric productivity [43,44]. Airlift reactors favor *Bacillus* production due to efficient oxygen transfer and low shear stress, being even more effective when coupled with foam removal systems [45]. Fluidized bed systems, inverse fluidized beds, and immobilized cell reactors also offer advantages, allowing biomass reuse and improved process stability, including for Rhamnolipid production [46].

Semi-continuous or repeated-batch processes allow partial medium withdrawal and replenishment, maintaining active biomass over multiple cycles while combining operational simplicity with productivity gains [47,48]. Fill-and-draw strategies have demonstrated significant improvements, with productivity increases of up to 163% compared to batch processes and 102% relative to fed-batch systems [47].

In situ product removal has become one of the most promising strategies, particularly when integrated with continuous or semi-continuous systems [49]. Foam fractionation is widely employed, exploiting the natural affinity of biosurfactants for the air–liquid interface [50,51]. For instance, *Bacillus atrophaeus* ATCC 9372 achieved high yields in airlift bioreactors coupled with foam columns [45]. Other approaches include solid adsorbents, ultrafiltration membranes, and liquid–liquid extraction using biocompatible solvents [49,52,53].

In summary, the integration of continuous or semi-continuous processes with ISPR represents a major advance in biosurfactant production intensification, reducing downstream costs, increasing productivity, and stabilizing processes, although challenges related to automation, large-scale economic feasibility, and strain stability remain [49,50].

While the use of crude or semi-purified extracts reduces production costs, the presence of residual impurities (such as free fatty acids, peptides, or unconverted substrates) can alter the Hydrophilic–Lipophilic Balance (HLB) of the system, leading to inconsistent emulsifying properties [54]. Moreover, for food applications, downstream processing is critical not only for functionality but for safety. Impurities in crude extracts may induce cytotoxicity or undesirable sensory changes, necessitating rigorous purification steps (such as ultrafiltration and solvent extraction) to meet food-grade standards and ensure batch-to-batch reproducibility [55].

## 5. Functional and Technological Properties in Foods

The food sector represents one of the most relevant areas of the global industry and has undergone significant transformations in recent years, driven by increasing consumer awareness. The demand for green products, those containing minimal or no chemical additives in their formulations, has stimulated the search for natural and sustainable alternatives for various applications. In this context, biosurfactants emerge as a promising alternative, as they can be obtained through renewable microbial routes, providing an environmentally viable option to current manufacturing processes and widely used synthetic surfactants. In addition to their physicochemical properties, such as emulsification, foam formation, antioxidant and antimicrobial activity, and surface tension reduction, among others [56,57], biosurfactants exhibit multifunctional potential in food systems.

The ability of biosurfactants to stabilize food formulations stems from their rapid adsorption kinetics at the oil–water interface. Due to their amphiphilic structure, they lower the interfacial tension significantly (often <30 mN/m for rhamnolipids and surfactin), facilitating droplet disruption during homogenization [58]. The reduction in interfacial tension is dose-dependent, with higher biosurfactant concentrations leading to greater reductions [59]. Stabilization is achieved through the Gibbs–Marangoni effect and the formation of a viscoelastic film that provides steric or electrostatic repulsion, preventing phenomena such as coalescence and Ostwald ripening [60,61].

In complex food matrices, biosurfactants interact dynamically with other macromolecules. In the presence of proteins, low-molecular-weight biosurfactants may compete for the interface (potentially displacing proteins) or form hydrophobic complexes that modify interfacial rheology [62]. Furthermore, in starchy foods, biosurfactants can form inclusion complexes with amylose, inhibiting starch retrogradation and improving texture stability [63].

*(a)*  *Emulsification*

Microbial biosurfactants are primarily classified based on their chemical structure into low-molecular-weight compounds, such as glycolipids, including rhamnolipids, sophorolipids, and lipopeptides, such as surfactin and lichenysin, as well as high-molecular-weight compounds, including polymeric surfactants, including liposan, emulsan, carbohydrate–lipid–protein complexes. This structural diversity directly influences their surface behavior [26].

The formulation of an effective emulsifier remains a challenge, as it depends on the complex interplay between the oil phase, the surfactant, and the co-surfactant. Surfactant efficiency is governed not only by physicochemical parameters, such as the Critical Micelle Concentration (CMC), the Critical Packing Parameter (CPP), and the HLB, which determines whether an emulsion will be oil-in-water (O/W) or water-in-oil (W/O), but also by processing conditions. The stability of the dispersed phase is critically dependent on the surfactant concentration and the input of mechanical energy (e.g., shear rate, homogenization pressure, or ultrasonication time) used to disrupt droplets and prevent coalescence [56].

In this context, biosurfactants demonstrate an excellent ability to reduce surface and interfacial tension, promoting the stabilization of oil-in-water emulsions. This property is essential for food products that rely on phase uniformity, such as sauces, mayonnaise, creams, and dairy beverages [57,64]. The feasibility of this application was validated by Araújo et al. [65] in mayonnaise-type sauces formulated with licuri oil, in which the biosurfactant acted as a co-emulsifier, ensuring physical stability and product safety for up to 240 days under refrigeration.

Among the most extensively studied compounds are rhamnolipids, sophorolipids, and lipopeptides. Classical studies on rhamnolipids have demonstrated that, when compared to traditional synthetic surfactants, biosurfactants offer greater thermal stability and biocompatibility, particularly under alkaline pH conditions, while also meeting clean-label demands [66,67,68].

Beyond emulsification capacity, certain classes of biosurfactants also influence air incorporation and the formation of stable foams, simultaneously improving the texture and stability of emulsions [2,69,70].

*(b)*  *Foaming and Texture Enhancing Agents*

In food production, aeration is a fundamental step, as the inclusion of air in the form of bubbles imparts distinct textures and sensory experiences to products. Air incorporation into food matrices significantly influences texture, resulting in increased firmness and softer, lighter characteristics that are pleasant to the palate [2].

Studies report the application of biosurfactants in the production of dairy products, bakery goods, and fermented foods, aiming to improve technological and sensory characteristics, ensuring enhanced texture and consistency [69,70]. The incorporation of a *Bacillus subtilis* SPB1 biosurfactant into cookie dough at a concentration of 0.1% relative to flour promoted notable improvements in dough texture. Similarly, the use of *B. subtilis* lipopeptides as emulsifying agents resulted in reduced firmness and improved dough consistency. Consequently, the inclusion of these biosurfactants led to the production of cookies with significantly softer textures [71,72].

In addition to these physicochemical effects, several biosurfactants also exhibit important biological activities, such as antioxidant and antimicrobial actions, which contribute to food stability, safety, and shelf-life extension [5,73].

### 5.1. Functional Properties

Biosurfactants stand out not only for their technological properties but also for their biological and functional activities, which contribute to food stability and safety, particularly antioxidant and antimicrobial actions. These properties enhance the potential of these compounds as multifunctional ingredients, capable of maintaining quality and extending the shelf life of food products [74].

*(a)*  *Antioxidant Activity*

Antioxidants are substances required to neutralize free radicals generated in the body during physiological processes. These highly reactive free radicals can cause severe damage known as oxidative stress or oxidative injury. Biosurfactants may act by altering the physicochemical properties of surfaces, preventing the adhesion of bacterial aggregates, and blocking oxidative chain reactions, thereby exhibiting antioxidant activity [75,76].

The antioxidant activity of biosurfactants is associated with the presence of functional groups such as hydroxyl, carboxyl, and amine groups in their molecular structures, which confer the ability to scavenge free radicals via hydrogen atom transfer (HAT) or single electron transfer (SET) mechanisms, including the DPPH radical (2,2-diphenyl-1-picrylhydrazyl), a key mechanism for interrupting oxidative reactions [76,77,78]. This function is particularly important in preventing lipid oxidation, which negatively affects the color, odor, and nutritional value of fat-rich foods. Recent studies indicate that glycolipids, such as sophorolipids, exert a protective effect in food emulsions and meat products (sausages, hamburgers, pâtés, and others), significantly reducing oxidation rates and preventing spoilage due to oxidative deterioration [64,74].

*(b)*  *Antimicrobial Activity*

Biosurfactants exert antimicrobial activity through mechanisms involving the integration of their amphiphilic moieties into microbial cell membranes. This interaction disrupts the phospholipid bilayer integrity, leads to alterations in membrane permeability, potentially causing metabolite leakage and cell lysis, inhibiting microbial adhesion to surfaces, and preventing subsequent biofilm formation [77,78]. This functional characteristic is highly relevant for controlling foodborne pathogens such as Listeria monocytogenes, Salmonella spp., and Escherichia coli. Thus, biosurfactants are consolidated as promising alternatives to synthetic chemical preservatives used in the food industry [79].

These bioactive properties have been explored through different approaches, ranging from incorporation into food formulations to application in bioactive packaging and coatings. In such systems, biosurfactants contribute synergistically to microbial load reduction and oxidation retardation. For example, when rhamnolipids and lipopeptides are incorporated into biopolymer films such as chitosan or starch, a combined effect of oxidative protection and microbial inhibition is observed. This dual benefit is crucial for extending shelf life and preserving fresh or minimally processed foods [80,81].

### 5.2. New Uses and Applications

*(a)*  *Fermented and Probiotic Beverages*

Biosurfactants have been extensively investigated as functional ingredients highly compatible with probiotic microorganisms, particularly in fermented beverages produced from milk, soy, and fruits. Their incorporation into these systems not only enhances the dispersion of nutrients and bioactive compounds but also contributes to improved foam stability and, critically, to the preservation of probiotic strain viability throughout the storage period [82].

Moreover, when produced by lactic acid bacteria (LAB), biosurfactants such as those derived from *Lactobacillus plantarum* and *L. paracasei* exhibit significant selective antimicrobial activity. This selectivity enables the control of harmful or pathogenic microorganisms without compromising the integrity of probiotic and fermentative cultures. These characteristics make biosurfactants ideal candidates for the development of probiotic beverages, offering combined functional and technological advantages [69,70].

*(b)*  *Bioactive Packaging*

Food preservation can be compromised by several factors, resulting in quality deterioration and sensory changes, including alterations in color and flavor. Microbial growth and oxidative reactions represent major challenges to maintaining food quality [83].

The potential of biosurfactants extends beyond the food matrix itself. The same properties that promote emulsion stability, antimicrobial activity, and oxidative control are equally valuable in the formulation of preservation systems. These compounds can be incorporated into biodegradable polymer matrices, such as chitosan, starch, and polylactic acid (PLA), to impart antioxidant, antimicrobial, and barrier properties [80,81].

Recent studies demonstrate that films containing rhamnolipids or surfactin significantly reduce microbial growth in fresh products and preserve visual and nutritional quality for longer periods [82]. Therefore, the development of new strategies is essential for maintaining food quality, particularly during storage. To overcome these challenges, the use of active packaging, primarily based on polymeric matrices with antioxidant properties, has been considered an effective tool [83]. In this context, bioactive packaging incorporating biosurfactants represents a promising frontier, combining natural preservation with environmental sustainability.

*(c)*  *Replacement of Synthetic Additives*

Innovation in food additive use is not limited to the substitution of synthetic compounds with natural alternatives, but also involves the application of microbial and biotechnological processes that enable the development of more sustainable ingredients with potential benefits to human health. Traditionally, food additives are widely applied in the industry to preserve, stabilize, and enhance the sensory and technological quality of processed products. These compounds encompass a broad range of functions, including colorants, preservatives, emulsifiers, thickeners, antioxidants, acidulants, pH regulators, and anti-caking agents, among others [84,85].

The multifunctionality of biosurfactants allows their application as natural substitutes for several synthetic additives, including emulsifiers, preservatives, and antioxidants. This substitution reduces environmental impacts and potential toxicological risks associated with artificial additives [74,86]. From an industrial perspective, challenges related to standardization, production costs, and scalability still exist; however, recent advances in bioprocessing using agro-industrial residues and microbial genetic engineering have been progressively mitigating these limitations [31]. Furthermore, economic feasibility studies and life cycle assessment (LCA) analyses indicate that biosurfactant production from renewable substrates may become competitive with synthetic surfactants in the medium term [85].

In addition to advances in production, biosurfactants exhibit technological and functional properties highly relevant to the formulation of stable, safe, and sustainable food products.

Beyond their technological role as additives for texture and stability, biosurfactants are increasingly recognized for their bioactive potential. This dual functionality paves the way for their application in the growing market of functional foods and nutraceuticals [27], as discussed in the following section.

## 6. Perspectives in Functional Foods and Nutraceuticals

According to the definition established by FAO/WHO in 2002, probiotics are live microorganisms that, when administered in adequate amounts, confer health benefits to the host. Probiotic strains are distinguished by their ability to synthesize a variety of bioactive metabolites, including biosurfactants. These bioactive substances play a decisive role in suppressing the proliferation of pathogenic microorganisms, thereby contributing to the maintenance of microbial homeostasis in the host [87].

Several studies have demonstrated that Lactic Acid Bacteria (LAB) are capable of synthesizing biosurfactants with promising applications in different food matrices and formulations. Considering that biosurfactants produced by probiotic LAB are capable of inhibiting adhesion and biofilm development on multiple abiotic interfaces, including equipment used in the food industry and processing areas, biosurfactant production emerges as an effective strategy to prevent microbial colonization by interfering with cellular adhesion. In the food industry, biofilm formation is a recurrent challenge, particularly in sectors such as culinary processing and bakery operations. Numerous bacteria colonize food contact surfaces and form biofilms, which are among the main causes of product deterioration during processing and storage [88,89].

LAB play an essential role in fermentative processes, contributing to the development of flavor, texture, and aroma in a wide variety of foods. In addition, these bacteria synthesize several metabolites during fermentation, including lactic acid, acetic acid, diacetyl, and volatile aromatic compounds, which significantly influence the sensory characteristics of fermented foods and beverages [90].

The production of biosurfactants by LAB, particularly species of the genera Lactobacillus and Lactococcus, which have been used in foods since ancient times and are recognized as GRAS (Generally Recognized as Safe), has received increasing attention [91]. Lactic acid bacteria such as *L. acidophilus*, *L. casei*, *L. rhamnosus*, and *L. fermentum* are recognized as important biosurfactant producers among probiotic bacteria. Biosurfactant synthesis by *Lactobacillus* species enhances their competitiveness against pathogenic microflora in the gastrointestinal tract [92,93,94]. LAB play critical roles in the fermentation of dairy and meat products, exerting a decisive influence on the quality and preservation of final products [95].

Meiguni et al. [96] evaluated 80 bacteria isolated from 70 dairy samples. Isolate F20, selected based on probiotic tests and identified by 16S rRNA sequencing as *Levilactobacillus brevis*, was identified as a promising candidate for biosurfactant production. Thin-layer chromatography (TLC) and FTIR analyses confirmed the presence of glycolipids and lipopeptides in the biosurfactant composition. The study demonstrated that the L. brevis F20 strain has significant potential as a biosurfactant producer for applications in the food industry.

Parvin et al. [97] investigated the antimicrobial and antibiofilm properties of biosurfactants produced by *Lactiplantibacillus pentosus* MSCIN-24 and *L. pentosus* MSCIN-25 (GenBank Acc. No.: MK397508) against pathogens associated with food spoilage and topical infections. The glycolipid biosurfactants exhibited broad-spectrum antimicrobial activity, with Minimum Inhibitory Concentration (MIC) and biofilm inhibition values ranging from 5 to 15 mg/mL, in addition to promoting cellular disruption. These findings indicate that such glycolipids possess significant potential as antimicrobial and antibiofilm agents against foodborne and topical pathogens.

## 7. Safety and Regulation

The successful incorporation of biosurfactants into the food market depends on overcoming critical barriers that extend beyond technical performance: rigorous assessment of safety for human consumption, compliance with the complex international regulatory landscape, and achievement of consumer acceptance. Failure in any of these aspects may represent an insurmountable obstacle.

### Toxicological and Microbiological Safety

Toxicological safety is the primary criterion for the approval of new food ingredients [98]. The evaluation of biosurfactants follows a tiered and systematic approach based on established principles, including acute toxicity, subchronic toxicity (28 or 90 day studies), genotoxicity, carcinogenicity, and reproductive toxicity assessments. For products derived from microbial metabolism, such evaluation is particularly critical to rule out adverse effects associated with molecular structure or residual contaminants [99,100].

Surfactin is one of the most extensively studied biosurfactants in terms of safety [101]. Genotoxicity assessment of Surfactin C consistently demonstrated negative results in the Ames Test (microbiological assay used to assess mutagenic potential), both in the presence and absence of metabolic activation and in the in vivo micronucleus assay in mice. Regarding acute oral toxicity, surfactin exhibits a favorable profile, with LD_50_ values exceeding 2.5 g/kg or even 5 g/kg, classifying it as a substance of very low toxicity. Additionally, developmental toxicity studies revealed no teratogenic effects, establishing a No Observed Effect Level (NOEL) of 500 mg/kg/day [102].

In vitro cytotoxicity studies further complement this evaluation. In Caco-2 cells, a human intestinal epithelial model, surfactin exhibited low cytotoxicity at concentrations up to 125 µg/mL after 48 h [103]. In normal human lung fibroblasts (WI-38), purified surfactin showed a safe dose (EC_100_) of 13.1 µg/mL and an IC_50_ of 112.4 µg/mL; however, conjugation with hyaluronic acid significantly improved its safety profile [104]. Other studies in skin cell models (fibroblasts and keratinocytes) also indicated the absence of significant cytotoxicity at concentrations relevant for topical applications. Collectively, these data suggest a robust safety profile for surfactin, particularly regarding genotoxicity and acute and developmental oral toxicity [105,106].

In contrast, Lichenysin, a lipopeptide biosurfactant produced mainly by Bacillus species, particularly *Bacillus licheniformis*, exhibits a toxicological profile that warrants attention [107]. In vitro studies using models relevant to intestinal exposure demonstrated significant cytotoxicity. The concentration of Lichenysin required to reduce cell viability by 50% (IC_50_) after 72 h was 16.6 µg/mL in Caco-2 cells (human intestinal epithelium) and 16.8 µg/mL in porcine ileum organoids. These findings raise concerns regarding the potential risk of food intoxication associated with elevated Lichenysin levels in contaminated products. Nevertheless, the compound exhibited low toxicity in human kidney cells (HEK293), with IC_50_ values exceeding 200 µg/mL. Data on genotoxicity and in vivo oral toxicity for Lichenysin remain scarce in the literature [108].

Sophorolipids exhibit distinct safety profiles depending on their acidic (ASL) or lactonic (LSL) forms [109]. Comparative studies suggest that the acidic form is generally less toxic than the lactonic form. In vitro assays showed that ASL had no visible effects on ovarian cancer cells or keratinocytes at concentrations where LSL exhibited toxicity. Moreover, ASL is considerably less hemolytic than the chemical surfactant Tween-80. Overall, sophorolipids appear to have a low in vitro toxicity profile, including in human renal cells [108,110].

Regarding rhamnolipids, toxicological evaluations have yielded variable results; however, recent studies using purified forms suggest a more favorable safety profile than previously assumed, particularly for mono-rhamnolipids. These compounds exhibited negligible toxicity in human keratinocytes (estimated LD_50_ > 600 µg/mL), being significantly less toxic than the synthetic surfactant SLES and di-rhamnolipids. Purification is essential to mitigate risks associated with production by *Pseudomonas aeruginosa*, although in vivo and genotoxicity data for purified compounds remain limited [111].

Biosurfactants derived from microorganisms considered safe (GRAS), such as lactic acid bacteria, are of particular interest. A glycolipopeptide isolated from Lactobacillus casei NM512 maintained cell viability above 79% at 100 µg/mL in MDA-MB-231 breast cancer cells [112]. Another glycolipopeptide from *Lactobacillus pentosus* NCIM 2912, at a concentration of 1 mg/mL, exhibited low cytotoxicity (>90% viability) in HEK293 (human kidney), L929 (mouse fibroblast), and HEP-2 (human epithelial) cell lines; however, the relevance of these data to direct human food safety remains limited [113].

The GRAS status of some biosurfactant producing microorganisms is advantageous but does not eliminate the need for compound specific toxicological testing. A comparative synthesis of these findings, focusing on key aspects relevant to oral human consumption safety, is presented in Table 2.

## 8. Global Regulatory Landscape and Consumer Acceptance

Navigating the complex regulatory landscape represents one of the most significant barriers to the commercial adoption of these compounds. A crucial distinction lies between the regulatory status of the producing microorganisms and that of the purified biosurfactant. Although strains such as Bacillus subtilis hold GRAS status in the United States or QPS (Qualified Presumption of Safety) status in the European Union, this safety recognition applies to the microorganism itself and does not automatically extend to the isolated molecule used as a food ingredient [114,115].

In the United States, the introduction of new substances intended for food use is overseen by the Food and Drug Administration (FDA). Two main regulatory pathways exist for the approval of new food ingredients: the Food Additive Petition (FAP) or recognition as GRAS. The GRAS pathway, which may be self-affirmed by the company or notified to the FDA for review, is based on demonstrating safety through “scientific procedures” or “experience based on common use in food” prior to 1958. For novel substances or those produced via new methods, such as many biosurfactants, demonstration through scientific procedures (peer-reviewed publications and toxicological studies) is the most likely route [116].

Despite the GRAS status of certain producing microorganisms, such as Bacillus subtilis, approval of purified biosurfactants as direct food ingredients in the United States remains limited. Specifically for sophorolipids, recent reviews confirm that they do not currently hold GRAS status granted or notified to the FDA [1].

However, it is essential to distinguish between food additive regulation (FDA) and regulation of substances used in agriculture whose residues may be present in foods (Environmental Protection Agency, EPA). For rhamnolipids, a significant regulatory milestone was achieved in 2004, when the EPA issued a final rule establishing an exemption from tolerance requirements (i.e., exemption from a maximum residue limit) for rhamnolipids in all food commodities. This exemption applies when rhamnolipids are used either as active ingredients (fungicides, insecticides) in agriculture or as inert ingredients (adjuvants, surfactants) in pesticide formulations [117].

On the other hand, this approval does not allow its use as an ingredient added directly to food for human consumption. Similarly, sophorolipids are on the EPA’s “Safer Choice” list for cleaning products (https://www.epa.gov/saferchoice, accessed on 22 October 2025), which also does not imply safety for ingestion.

In the European Union, the introduction of new food ingredients is strictly regulated, primarily through the Food Additives Regulation (EC) No. 1333/2008 and the Novel Food Regulation (EU) 2015/2283. A food additive can only be used if it is included in the Union’s positive list, following a safety assessment by the EFSA (European Food Safety Authority) and authorization by the European Commission [118].

The current situation for specific biosurfactants in the EU reflects the absence of approvals for direct food use. Sophorolipids, for example, are not approved as food additives under Regulation (EC) No. 1333/2008. It is important to note that sophorolipids are approved for use in cosmetics in the EU and are registered under REACH (Registration, Evaluation, Authorisation and Restriction of Chemicals), but these authorizations do not extend to their use as ingestible food ingredients [119].

In Brazil, the regulation of food additives is under the responsibility of the Agência Nacional de Vigilância Sanitária (ANVISA). For a new substance to be used, it must be evaluated for toxicological safety and included in the positive lists of authorized additives, as established by RDC No. 778/2023 (https://www.in.gov.br/web/dou/-/resolucao-rdc-n-778-de-1-de-marco-de-2023-468499613, accessed on 15 October 2025). A petition from the interested party is generally required to request the inclusion of a new ingredient. To date, no explicit approval by ANVISA has been identified for the use of purified biosurfactants, such as surfactin or sophorolipids, as direct food additives.

These regulatory constraints highlight the urgent need for harmonized international frameworks and robust toxicological datasets to support the approval of biosurfactants for food applications.

### Consumer Acceptance

Even after overcoming safety and regulatory barriers, consumer acceptance represents the final hurdle. As these are novel ingredients, there is no consolidated perception of consumption, and their success will depend largely on the framing and narrative of their presentation. Acceptance is articulated around a duality: alignment with the Clean Label trend versus the unease caused by technical nomenclature and microbial origin. On the one hand, biosurfactants align with the demand to replace synthetic additives, supported by a positive narrative of production via fermentation and residue valorization (upcycling), which reinforces the concepts of sustainability and circular economy valued by consumers [120,121].

On the other hand, significant perceptual barriers exist. The first relates to nomenclature: scientific terms such as “sophorolipid” or “rhamnolipid” may sound artificial and trigger food neophobia, being perceived as “chemical” despite their biological nature [122]. The second barrier concerns origin, as the idea of a purified microbial-derived ingredient may generate hesitation. This resistance becomes critical if the producing strains are genetically modified (GMOs) to enhance industrial efficiency. The low acceptance of GMO-derived ingredients, particularly in markets such as Europe, creates a direct conflict between technical production feasibility and consumer preference. Commercial viability will therefore depend on transparent communication that translates the biotechnological origin into a clear and safe value proposition [123,124,125].

## 9. Sustainability and Circular Economy

**Figure 4:**
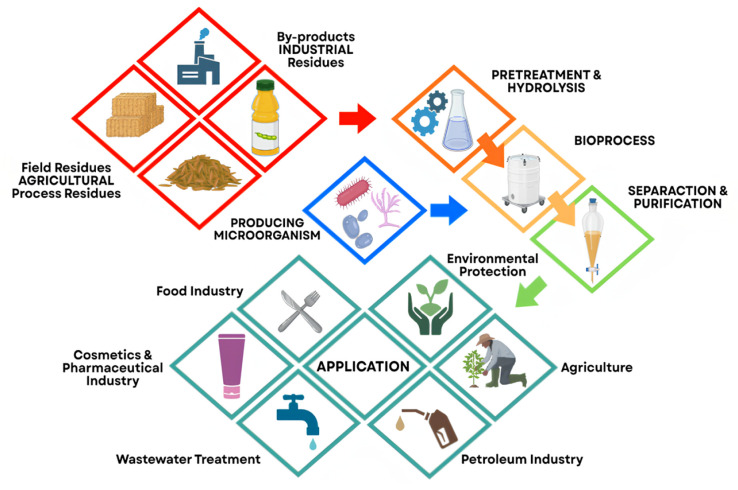
Sustainable production of biosurfactants using agro-industrial residues and by-products. Source: Adapted from Vucurovic et al. [28], published by MDPI, 2024.

The growing demand for more sustainable surfactants has placed biosurfactants at the center of circular economy initiatives, particularly due to their biodegradability and potential to replace petrochemical surfactants as a sustainable alternative [126]. The recent literature highlights that the use of residual feedstocks and agro-industrial by-products (such as oilseed cakes, molasses, fruit residues, and effluents containing assimilable carbon) represents a promising route to reduce both raw material costs and the environmental footprint of microbial production (Figure 4). Review articles and experimental studies have shown that these “second-generation feedstocks” can supply sufficient carbon and lipids for cost-effective fermentations; however, they require pre-treatment strategies and strict control of compositional variability to ensure reproducibility and yield [28,31].

Integrated environmental assessments have become essential to demonstrate the actual environmental advantages of biosurfactants over their synthetic counterparts. Life Cycle Assessment (LCA) studies applied at early stages of process development indicate that the main environmental hotspots are often associated with energy consumption during aeration/fermentation, downstream operations (concentration and purification), and the origin of the substrate. Therefore, production routes that combine residual feedstocks with process intensification and in situ product removal strategies tend to exhibit more favorable environmental profiles, provided that the major impact points identified by LCA are adequately controlled. These findings have encouraged the incorporation of LCA and cost analyses already at the process design stage (eco-design), enabling technology choices guided by realistic trade-offs between environmental impact and economic feasibility [127,128].

From a circular economy perspective, the development of value chains that integrate co-products is critical to improving economic viability and resource efficiency. Well-designed processes can generate complementary streams, such as residual biomass fractions converted into biogas or biofertilizers, residual lipids recovered for other bioproducts, and the biosurfactant itself used as an input for industrial cleaning, soil remediation, or green cosmetic formulations. Recent reviews highlight cases in which the integration of waste valorization and microbial production increases the overall value added of the system and contributes to closing material loops among agricultural, food, and chemical industries [28,129].

Process intensification techniques have been investigated with the aim of increasing biosurfactant productivity and concentration during fermentation while simultaneously reducing energy consumption and costs associated with purification steps. Strategies such as foam fractionation, adsorption in packed beds, ultrafiltration, and liquid–liquid extraction with appropriately selected solvents have shown promising results, particularly when combined with low-cost substrates, thereby contributing to more efficient and sustainable processes [45,128].

However, large-scale application requires the development of an integrated and economically viable process grounded in the principles of waste reduction, reuse, and recycling. Industrial production still faces technical and economic challenges, including variability and pre-treatment requirements of residual feedstocks, high energy demand in aerobic processes, the lack of standardized data for comparative environmental assessments, and financial uncertainties associated with scale-up. In addition, factors such as strain stability, automated process control, and regulatory and market acceptance remain barriers that require further investigation and pilot-scale validation [31,128,130].

## 10. Conclusions

Microbial biosurfactants represent a promising and multifunctional alternative to synthetic surfactants used in the food industry, combining technological, functional, and environmental properties of great relevance to the sector. Recent advances in bioprocessing, including the use of agro-industrial residues, genetic engineering of strains, continuous production systems, and in situ product removal strategies, have contributed to cost reduction and efficiency gains, bringing the production of these compounds closer to industrial feasibility.

From a technological perspective, biosurfactants demonstrate high effectiveness as emulsifiers, texture-modifying agents, and foam stabilizers, in addition to exhibiting antioxidant and antimicrobial activities that favor the extension of food shelf life. Their incorporation into fermented beverages, probiotic systems, and bioactive packaging highlights their potential as functional ingredients capable of combining sensory quality, stability, and microbiological safety.

Lactic acid bacteria and other GRAS species emerge as particularly relevant sources due to the production of biosurfactants with favorable bioactive profiles and compatibility with food systems. However, the application of these compounds remains limited by regulatory challenges, the lack of standardized toxicological frameworks, and high production costs. Consumer acceptance, in turn, will depend on communication strategies that emphasize their natural origin, biodegradability, and contribution to clean-label formulations.

From an environmental standpoint, the integration of biosurfactant production with circular economy principles and the use of low-impact feedstocks reinforces their role in the transition toward more sustainable food systems.

In summary, although technical, economic, and regulatory barriers persist, current scientific evidence indicates that biosurfactants have the potential to become strategic ingredients for the development of safer, more functional, and more sustainable foods. Concurrent progress in research, regulation, and industrial innovation will be decisive for their large-scale adoption in the coming years.

## Figures and Tables

**Table 1:** Types of agro-industrial residues used as carbon and nitrogen sources for biosurfactant production, indicating origin, predominant composition, producing microorganism, biosurfactant class, and yield obtained.

Type of Waste	Origin/Sector	Main Composition	Producing Microorganism	Type of Biosurfactant	Approximate Yield	Ref.
Sugar beet molasses	Sugar industry	Complex lignocellulosic compounds	*Pseudomonas putida* KT2440	Rhamnolipids	5.40 g·L^−1^	[18]
Sugarcane molasses	Sugar and ethanol industry	Total sugars, sucrose and glucose	*Bacillus subtilis* UFPEDA 438	Surfactin	0.008187 g·L^−1^·h^−1^	[19]
Waste frying oil	Food/ Domestic	Triglycerides and fatty acids	*Pseudomonas syringae* pv *tabaci*	Lipopeptides	2.70 g·L^−1^	[20]
Whey	Dairy products and milk derivatives	Lactose and soluble proteins	*Limosilactobacillus fermentum* ACA-DC 0183	glycolipoprotein	0.29 g·L^−1^	[21]
Crude glycerol	Biodiesel industry	glycerol and methanol	*Pseudomonas fluorescens* DR54	Viscosinamide (cyclic lipopeptide)	0.57 g·L^−1^	[22]
Palm oil residues	Palm oil refinery	PFAD—Palm Fatty Acid Distillate e FAME—Fatty Acid Methyl Ester	*P. aeruginosa* PAO1	Rhamnolipid	1.07–2.11 g·L^−1^	[23]
Cassava waste	Cassava starch and flour industry	Cassava processing wastewater: starch, reducing sugars, nitrogen compounds, traces of cyanides.	*Bacillus subtilis* UCP 0999	lipopeptide	2.67 g·L^−1^	[24]
Concentrated pineapple peel juice	Pineapple processing agro-industry	Total sugars	*Bacillus subtilis* LMA-ICF-PC 001	lipopeptide	1.28 g·L^−1^	[17]
Corn Steep Liquor	Corn processing industry	carbohydrates, amino acids, peptides, vitamins and minerals	*Aneurinibacillus aneurinilyticus* (CECT 8489)	lipopeptide	6.0 g·L^−1^	[25]

**Table 2:** Comparative summary of the toxicological profiles of selected biosurfactants, focusing on aspects relevant to human oral consumption safety. LD_50_: lethal dose 50%; NOEL: No Observed Effect Level; Caco-2: human colorectal adenocarcinoma cell line; Org.: organoids; IC_50_: inhibitory concentration 50%.

Biosurfactant	Genotoxicity (Oral/Relevant)	Acute Oral Toxicity (LD_50_)	Developmental Toxicity (Oral)	Intestinal Cytotoxicity (Caco-2/Org.)	General Profile (Ingestion)
Surfactin	Low Risk (Negative)	Low Risk (>2500 mg/kg)	Low Risk (NOEL 500 mg/kg/d)	Low Risk	Promising
Lichenisin	ND	ND	ND	Concerning (low IC_50_)	Concerning
Sophorolipid Acid	ND	ND	ND	ND	Low Potential
Mono-Rhamnolipid	ND	ND	ND	ND	Low Potential
Di-Rhamnolipid	ND	ND	ND	ND	Low Potential

Risk classification “ND” indicates no data available.

## Data Availability

No new data were created or analyzed in this study. Data sharing is not applicable to this article.
